# The Role of Bile Pigments in Health and Disease: Effects on Cell Signaling, Cytotoxicity, and Cytoprotection

**DOI:** 10.3389/fphar.2012.00136

**Published:** 2012-07-13

**Authors:** Jaime Kapitulnik, Mahin D. Maines

**Affiliations:** ^1^Laboratory of Drug Metabolism, Institute of Drug Research, School of Pharmacy, Faculty of Medicine, The Hebrew University of JerusalemJerusalem, Israel; ^2^Biochemistry and Biophysics, University of Rochester School of MedicineRochester, NY, USA

Degradation of heme involves its conversion to biliverdin by heme oxygenase followed by reduction of biliverdin to bilirubin by biliverdin reductase. There is ample evidence for the role of heme oxygenase in protecting cells from the toxic effects of heme, as well as for the pleiotropic functions of biliverdin reductase in cell signaling and regulation of gene expression. This enzyme plays a major role in glucose uptake and the stress response. Bilirubin has been shown to behave as a “double-edged sword.” It can exert either cytotoxic or cytoprotective effects, depending on the blood and/or tissue concentration of its free fraction, the nature of the target cell or tissue, and the cellular redox state. The central nervous system is particularly sensitive to the neurotoxic effects of bilirubin. Its antioxidant effect is the basis for the proposed cardioprotective effect of bilirubin in humans with moderate hyperbilirubinemia, as is the case in subjects with the Gilbert syndrome.

This Research Topic forum is intended to serve as a platform for updating information and presenting advances in basic and clinical research in the above and related subjects. The topic is discussed by leading experts in the field of bile pigments, and presented in 15 Reviews, 3 Original Research articles and 1 Opinion article. It covers important aspects related to the enzymes involved in the heme catabolic pathway: the role of heme oxygenase in inflammation and fibrosis (Lundvig et al., 2012) as well as in atherosclerosis (Araujo et al., 2012) and immune-mediated inflammatory diseases (Larsen et al., 2012), the regulation of cell signaling by biliverdin reductase and its peptide fragments (Gibbs et al., 2012), and the regulation of bilirubin clearance (Bock, 2011).

The role of glial cells and inflammation in bilirubin neurotoxicity (Brites, 2012) and the transport and metabolism of bilirubin at blood-brain interfaces and neural cells (Gazzin et al., 2012) illustrate the complex nature of bilirubin-induced brain damage. The effects of bilirubin vary with age (Dennery, 2012), and metalloporphyrins have been suggested to reduce excessive hyperbilirubinemia and brain damage in newborns (Schulz et al., 2012).

The regulatory properties of bile pigments and the role of biliverdin reductase in mediating their antioxidative (Jansen and Daiber, 2012) and anti-inflammatory effects (Wegiel and Otterbein, 2012), and their role in aging and age-related diseases (Kim and Park, 2012), are only part of the known protective functions of bile pigments. Bilirubin displays antiviral activity (Santangelo et al., 2012; Schmidt et al., 2012), ameliorates renal hemodynamics and blood pressure in an animal model of hypertension (Stec et al., 2012), and has beneficial effects in pulmonary and vascular diseases (Ryter, 2012). The protective effects of bilirubin in the vasculature include inhibition of neointima formation and reduction of vascular smooth muscle cell proliferation and migration (Peyton et al., 2012). In microvascular endothelial cells, low (“physiological”) bilirubin concentrations induce apoptosis, which is exacerbated under hyperglycemic conditions. Endothelial cells of the blood-brain barrier are particularly sensitive to these effects of bilirubin (Kapitulnik et al., 2012).

## Conclusion

Bilirubin, which has been considered for decades to be a toxic waste product of heme catabolism, is now recognized as an endogenous cytoprotective compound at low (“physiological”) concentrations. However, its protective effects have been demonstrated *in vitro*, mainly in peripheral tissues, while its neurotoxicity remains unchallenged.

Although moderately elevated plasma bilirubin levels (as those of subjects with Gilbert syndrome) have been shown in retrospective and prospective clinical studies to be associated with a decreased risk of cardiovascular diseases (Vitek, 2012), there is still no general concensus in applying this knowledge in manipulating bilirubin levels for the prevention of cardiovascular and associated diseases. Stimulation of biliverdin reduction and/or inhibition of bilirubin conjugation are highly questionable with regards to their safety in humans, particularly in the scenario of a life-long treatment. We hope that the work presented in this Research Topic will stimulate further basic and clinical research in the area of bile pigments and its pathological as well as therapeutic implications.
